# The epidemiology of *Stenotrophomonas maltophilia* and *Achromobacter xylosoxidans* infections

**DOI:** 10.1017/ash.2024.11

**Published:** 2024-02-02

**Authors:** Shani Zilberman-Itskovich, Edward Cohen, Leonid-Arie Ploshansky, Alexander Yusupov, Ruth Bouganim, Katie H. Jaffe, Dror Marchaim

**Affiliations:** 1 Shamir (Assaf Harofeh) Medical Center, Zerifin, Israel; 2 Faculty of Medicine, Tel Aviv University, Tel-Aviv, Israel

## Abstract

*Stenotrophomonas maltophilia* and *Achromobacter xylosoxidans* are emerging nosocomial, non-glucose fermenting, Gram-negative pathogens. In this nested case-control trial, independent predictors for *S. maltophilia* infections were hemodialysis and recent antibiotic usage (overall), while recent usage of fluoroquinolones, was independently associated with *A. xylosoxidans* infections. Infections were independently associated with multiple worse outcomes.

## Introduction

As declared by the World Health Organization (WHO), antimicrobial resistance is one of the biggest challenges and threats in modern Medicine.^1^ Extensively drug-resistant organisms (XDRO), specifically non-glucose fermenting XDR Gram-negatives (eg, *Pseudomonas aeruginosa*, *Acinetobacter baumannii*), pose an urgent worldwide concern to human health, mainly due to serious lack in therapeutic options.^1^ This leads to delay in initiation of appropriate antimicrobials,^2^ which is the strongest modifiable predictor for mortality in septic shock.^2^

Two additional non-glucose fermenting XDR Gram-negatives, which frequently display XDR phenotypes,^3,4^ are *Stenotrophomonas maltophilia* and *Achromobacter xylosoxidans*.^4,5^ These microorganisms were perceived in the past as commensals,^3^ but are increasingly reported as causative pathogens, associated with serious infections, particularly among intensive care unit (ICU) patients.^4,5^ The commonest reservoir for these organisms is the respiratory tract, and the commonest infectious syndromes are pneumonia (including ventilated-associated pneumonia) and blood stream infections [BSI]).^5^ These pathogens have intrinsic mechanisms of resistances (both inducible and constitutive production^3^) to most antimicrobial agents,^3–5^ including reports of pan-resistant isolates.^4–6^ Due to baseline characteristics,^4–6^ and presumable frequent delays in initiation of appropriate therapy,^2^ devastating outcomes were reported from non-controlled case-series analyses, with high morbidity and mortality rates.^4,5^ The aim of this study was to explore the descriptive epidemiology, the independent predictors, and the outcomes, of *S. maltophilia* and of *A. xylosoxidans* invasive infections.

## Methods

Retrospective, observational, matched nested case-control study was conducted for calendar years 2011–2022, at Shamir (Assaf Harofeh) Medical Center (895-bed university-affiliated acute-care facility), Israel. Data were obtained from all available records, and mortality data were retrieved from the Israeli Ministry of Interior records. The local ethic (“Helsinki”) committee approved the study prior to its initiation.

All adult (>18 years) hospitalized patients with *S. maltophilia* or with *A. xylosoxidans* bloodstream infection (BSI), per established BSI definition,^7^ were enrolled. The time trend of infections over the years were analyzed by the Cochran-Armitage Test. Each BSI case was matched to three control (uninfected) patients, based on pre-defined matching criteria (depicted in order of importance): 1) ‘time at risk’ (number of days from admission to infection, ±5 days), 2) type of admitting ward (eg, Medicine, Surgery, ICU), and 3) age (per decade). Matched analyses of predictors to *S. maltophilia* and of predictors to *A. xylosoxidans* BSI, and non-matched outcomes analyses (nine different morbidity and mortality outcome parameters), were queried by logistic or Cox regressions, respectively.

## Results

A total of 66 patients with *S. maltophilia* BSI and 8 patients with *A. xylosoxidans* BSI were enrolled. Twenty-three patients (31%) had a polymicrobial infection. There were no significant elevations in the incidences of *S. maltophilia* BSI (p for trend 0.45) and of *A. xylosoxidans* BSI (p for trend 0.57) during the 12-year study’s period. Five patients with *S. maltophilia* BSI could not be matched to control patients due to extreme length of ‘time a risk’ and were excluded from the analyses that compared cases to controls (Table 1), but not from the case-series descriptive analysis. Patients with *S. maltophilia* BSI or *A. xylosoxidans* BSI had a median age of 74 years (interquartile range [IQR] 67–82 years), 67% were males, and 16% were chronic residents of long-term care facilities (LTCF).

**Table 1.** Predictors and outcomes of patients with *Stenotrophomonas maltophilia* bloodstream infections (BSI), Shamir Medical Center, 2011–2020

Parameters		Cases No. (valid percent^1^)	Controls No. (valid percent^1^)	OR (CI-95%)	*P*-value
**Demographics**					
Age (years), median (IQR)		74 (68–86)	74 (67–82)	0.94	
Male gender		41 (67%)	103 (56%)	0.63 (0.34–1.16)	0.13
LTCF resident		10 (16%)	22 (12%)	1.44 (0.64–3.23)	0.38
**Background medical conditions and statuses**					
**Dependent functional status at admission**		**38 (62%)**	**71 (39%)**	**2.6 (1.44**–**4.74)**	**0.001**
Reduced cognition/consciousness upon admission		20 (33%)	56 (31%)	1.12 (0.6–2.06)	0.75
Chronic heart failure		29 (48%)	76 (42%)	28 (0.71–2.28)	0.41
Diabetes mellitus		32 (53%)	89 (49%)	1.17 (0.65–2.08)	0.61
**Chronic renal disease**		**31 (51%)**	**48 (26%)**	**2.91 (1.59**–**5.3)**	**<0.001**
Chronic lung disease^2^		13 (21%)	50 (27%)	0.72 (0.36–1.44)	0.35
Malignancy^3^		18 (30%)	58 (32%)	0.9 (0.48–1.7)	0.75
Chronic skin ulcer^4^		10 (16%)	31 (17%)	0.96 (0.44–2.1)	0.92
Charlson’s scores	Weighted Index Comorbidity	4.5 ± 2.8	3 (2–6)	0.23	
	Combined Condition Score	7.5 ± 3.2	7 (5–9)	0.32	
	10-Year Survival Probability	0 (0–21)	0 (0–21)	0.3	
Immunosuppression	**Recent (3 months) exposure to glucocorticoids** ^**5**^	**7 (12%)**	**43 (24%)**	**0.42 (0.18**–**1)**	**0.04**
	Recent (3 months) exposure to immunosuppressants and/or immunomodulators^6^	3 (5%)	6 (3%)	1.53 (0.37–6.3)	0.7
	**Overall Immunosuppression** ^**7**^	**7 (12%)**	**49 (27%)**	**0.36 (0.15**–**0.83)**	**0.014**
**Previous exposure to healthcare system**					
Recent carrier of a multidrug resistant pathogen^8^		14 (23%)	50 (27%)	0.79 (0.4–1.56)	0.5
**Recent (<3 months) hospitalization**		**40 (66%)**	**86 (47%)**	**2.38 (1.28**–**4.41)**	**0.005**
Recent (<3 month) stay in ICU		27 (44%)	72 (39%)	1.22 (0.68–2.2)	0.5
**Hemodialysis^9^**		**16 (26%)**	**11 (6%)**	**5.56 (2.41**–**12.81)**	**<0.001**
Invasive procedure in the past 6 months^10^		38 (62%)	89 (49%)	1.73 (0.95–3.13)	0.07
Permanent devices^11^		39 (64%)	111 (61%)	1.15 (0.63–2.1)	0.65
Recent mechanical ventilation (in prior 3 months)		**34 (56%)**	**55 (30%)**	**2.91 (1.6**–**5.28)**	**<0.0001**
**Number of days on mechanical ventilation, mean ± SD**		**10 ± 15**	**0 (0**–**1)**	**0.001**	
**Recent exposure to antimicrobials (in the past 3 months) for >48 hours duration**					
**Recent (previous 3 months) antibiotic use (overall)**		**52 (85%)**	**116 (63%)**	**3.34 (1.55**–**7.2)**	**0.001**
**Time from last Antibiotic use,** ^**12**^ **(days), median (range)**		**0 (0**–**0.8)**	**18 (3**–**60)**	**<0.001**	
Penicillin with/out beta-lactamase inhibitor		24 (39%)	61 (33%)	1.3 (0.71–2.36)	0.39
**Cephalosporin**		**38 (62%)**	**85 (46%)**	**1.91 (1.05**–**3.45)**	**0.032**
Carbapenem		19 (31%)	36 (20%)	1.85 (0.96–3.55)	0.063
Fluoroquinolone		17 (28%)	33 (18%)	1.76 (0.89–3.45)	0.1
Vancomycin		20 (32%)	40 (22%)	1.74 (0.92–3.31)	0.09
**Tigecycline**		**3 (5%)**	**1 (1%)**	**9.41 (0.96**–**92.26)**	**0.049**
Colistin		3 (5%)	9 (5%)	1 (0.23–3.82)	>0.99
Aminoglycoside		3 (5%)	14 (8%)	0.62 (0.17–2.25)	0.57
Metronidazole		18 (30%)	34 (19%)	1.83 (0.94–3.57)	0.07
**Outcomes**					
Died during hospitalization		**28 (45.9)**	**28 (15.3)**	**4.7 (2.47**–**8.95)**	**<0.001**
30 days mortality		**27 (44.3)**	**37 (20.2)**	**3.13 (1.68**–**5.83)**	**<0.001**
90 days mortality		**30 (49.2)**	**47 (25.7)**	**2.8 (1.53**–**5.11)**	**0.001**
Among survivors of the index hospitalization only	Functional status deterioration	16 (48.5)	96 (60.4)	0.62 (0.29–1.31)	0.21
	Discharge to LTCF after being admitted from home	11 (33.3)	61 (39.9)	0.75 (0.34–1.67)	0.48
	Re-admission in 3 months	**24 (72.7)**	**72 (45.3)**	**3.22 (1.41**–**7.37)**	**0.004**
	Invasive procedure^13^ in 3 months	**18 (54.5)**	**48 (30.4)**	**2.75 (1.28**–**5.91)**	**0.008**

Note. IQR = interquartile range; OR = odds ratio; CI = confidence interval; MIC = minimal inhibitory concentration; LTCF = long-term care facility; ICU = intensive care unit; SD = standard deviation; TNF = tumor necrosis factor.

1 Valid percent implies the percent after removing missing values from the denominator.

2 Chronic lung disease refers to bronchiectasis, chronic obstructive pulmonary disease (COPD), asthma, restrictive lung disease.

3 Malignancy refers to a patient that had in the past or currently having a malignancy, hematological or solid-organ.

4 Chronic skin ulcers refer to lower limb diabetic foot wounds, decubitus ulcers, dwelling wound surrounding PEG insertion, surgical-site wounds, wounds surrounding catheters. Wounds involving soft tissue/mucosa without skin involvement (eg, in mouth, GI tract, genital-urinary tract) were not included.

5 Systemic glucocorticoid/steroid use of at least three doses in the past month.

6 Immunosuppressants and/or immunomodulators includes for example anti-TNF (infliximab [Remicade], adalimumab [Humira], certolizumab pegol [Cimzia], golimumab [Simponi], etanercept [Enbrel]) and others.

7 Immunosuppression include any of the following: 1) Neutropenia at culture date (<500 neutrophils/mm^3^), 2) glucocorticoids exposure in the past month, 3) chemotherapy in the past 3 months, 4) radiotherapy, 5) post-transplantation of any kind, 6) anti-TNF therapy in past 3 months, or 7) HIV carrier.

8 Isolation in the past 6 months of any of the following: *Staphylococcus aureus* resistant to oxacillin (MRSA), ampicillin and/or vancomycin resistant enterococci (VRE), Enterobacterales which is resistant to any 3^rd^ or 4^th^ generation cephalosporin (ESBL), Enterobacterales with meropenem MIC > 1 (CRE), *Acinetobacter baumannii*, or *Pseudomonas aeruginosa*.

9 Hemodialysis units, weekly visits at sub-specialty clinics.

10 Endoscopies, biopsies, permanent central line insertions, percutaneous interventions, and any type of surgery, brain/cardiac catheterization, intubation and mechanical ventilation.

11 Permanent/chronic internal foreign devices that were placed at least 48 hours prior to bacterial isolation from blood culture, such as any drain or catheter are included (dialysis Perm-A-Cath). Internal foreign devices such as heart valves, prosthetic joints, pacemakers, subcutaneous insulin pumps were not included. Empiric regimen = Antibiotic treatment given 2 days prior to 3 days following culture date; Consolidative (main) therapeutic treatment = antibiotic therapy given 3 days to 14 days following the culture date.

12 Time from last antibiotic use refers to the days from patient’s last antibiotic day to the day of the index bacterial culture.

13 Invasive procedure includes any surgery, endoscopy, biopsy, percutaneous intervention, catheterization and intubation.

Of the *S. maltophilia* offending isolates, six (9.8%) were resistant to trimethoprim/sulfamethoxazole (TMP/SMX), three (4.9%) to levofloxacin, and four (6.6%) to tigecycline. Among the *A*. *xylosoxidans* isolates, one strain (12.5%) was resistant to TMP/SMX, seven (87.5%) to levofloxacin, and all isolates were susceptible to meropenem.

The Table displays the univariable comparisons between 61 *S. maltophilia* BSI and 183 matched controls. Patients with *S. maltophilia* BSI were more likely to be dependent in their functional status upon admission, be hemodialysis dependent, with higher rates of recent hospitalizations, and mechanical ventilation (Table 1). Patients who developed *S. maltophilia* BSI had higher rates of recent exposure to antibiotics (overall), ie, specifically to cephalosporins and to tigecycline. The time from last antibiotic use, was also significantly shorter compared to controls. In multivariable analysis, the only independent predictors to *S. maltophilia* BSI remained hemodialysis (aOR = 3.6, CI-95 = 1.3–10.2), and recent exposure to (any) antibiotics (aOR = 4.1, CI-95 = 1.4–12.5).

Outcomes were significantly worse among patients with *S. maltophilia* BSI (Table 1). In separate multivariable models, *S. maltophilia* BSI remained independently associated with in-hospital mortality (aOR = 7.4, CI-95 = 3.2–18.4), 30- and 90-day mortality (aOR = 3.2, CI-95 = 1.5–6.9, and aOR = 2.7, CI-95 = 1.3–5.7, respectively), and with readmission in the following three months (aOR = 3.2, CI-95 = 1.3–9.0).

Eight *A. xylosoxidans* BSI were matched to 24 matched controls. Patients with *A*. *xylosoxidans* BSI had median length of stay from admission to infection of 7 days (IQR 5–14 days). Compared to controls, patients with *A*. *xylosoxidans* BSI had significant shorter duration since their last hospitalization (20 days (IQR 5–77) vs. 100 days (IQR 27–460), *p* = 0.04), had significant higher number of days on mechanical ventilation (146 days (IQR 16–276) vs. 1 day (IQR 1–7), *p* = 0.01), and had elevated rates of recent exposure to fluoroquinolones (63% vs 13%, *p* = 0.01). In multivariable model, the only independent modifiable predictor to *A*. *xylosoxidans* BSI, remained recent exposure to fluoroquinolones (aOR = 7.1, CI-95 = 1.01–59.1). Outcomes of patients with *A*. *xylosoxidans* BSI were not significantly worse compared to matched controls.

## Discussion

The epidemiological burden and significance associated with nosocomial XDR Gram-negative infections is substantial, primarily due to lack of effective therapeutics, specifically infections resulting from non-glucose fermenting XDR isolates.^3^ The reported incidences of *S. maltophilia* and *A. xylosoxidans* infections from multiple hospitals are rising,^4–6^ with devastating outcomes reported from few case-series analyses.^4,5^ In this nested case-control trial, we explored the epidemiology of 66 *S. maltophilia* infections and of 8 *A. xylosoxidans* infections, for a 12-year period (2011–2022). In order to avoid classification bias and the inclusion of asymptomatic carriers as cases,^8,9^ only patients with BSI (per established definition^7,10^) were enrolled as cases. The incidences of *S. maltophilia* infections and of *A. xylosoxidans* infections, had not increased at SMC during the study.

Each BSI case was matched to three control patients, per strict matching criteria, determined a-priori, in accordance to established recommendations,^11^ in order to power the small sample size calculations. per multivariable analyses, the independent predictors for *S. maltophilia* BSI were chronic hemodialysis and recent antibiotic usage (of any class) in the previous three months. Previous recent usage of fluoroquinolones was the only independent predictor for *A. xylosoxidans* BSI, with wide confidence intervals for the models’ adjusted odds ratios, due to the low sample sizes. Nearly all patients who developed *S. maltophilia* infections (85%), or *A. xylosoxidans* infections (88%), had recent exposure to antimicrobials, implying that selective pressure is the main promotor for emergence of these resistant strains in hospitals. This might also imply that patient-to-patient transmission was not the common mode of transmission or dissemination at SMC during 2011–2022 of these pathogens, but this needs to be trialed specifically.

There were various worse outcomes, which were independently associated with *S. maltophilia* infections. Mortality rates (ie, in-hospital mortality, 30-day mortality, 90-day mortality) and rates of readmissions in the following three months among those who survived the index hospitalization, were all independently associated with *S. maltophilia* BSI. In previous analyses, *S. maltophilia* “colonization” was not associated with worse clinical outcomes.^6^ This further highlights the importance of the controlled design executed herein, and the fact that only ‘true’ BSI cases were enrolled as cases. In additional analyses who tried to enroll as cases, patients with *S. maltophilia* or *A. xylosoxidans* “clinical infection” (various definitions applied), infections were also statistically associated with worse outcomes.^4,5^ The associations of *A. xylosoxidans* BSI with increased mortality and readmission rates were evident in our univariable analyses, but due to the strict case definition criteria and the low sample size, it did not reach significance per multivariable models.

The main limitations of the study are the small sample of cases, specifically of the *A. xylosoxidans* cases. As previously mentioned, three control patients were matched to each case, in order to somewhat abate this limitation. In addition, the study has multiple inherent limitations associated with its retrospective chart review-based design, from a single Israeli center. The conclusions of this study could not be generalized nor extrapolated to other centers or to ambulatory settings (eg, patients with cystic fibrosis), without conducting additional detailed investigations. However, as far as we know, this is one of the largest analyses ever published in this evolving epidemiological field, which was executed with controlled design.

To conclude, *S. maltophilia* and *A. xylosoxidans* invasive infections were identified among 66 and 8 patients with prolonged hospitalization, during the years 2010–2020. The incidences are not rising at SMC over the years, but the infections are associated with serious and devastating outcomes. Selective pressure seems to promote infections, while in opposite to other XDR ICU non-glucose fermenting Gram-negative pathogens,^12^ this is the major suspected mode for emergence of these inherently resistant isolates. Independent predictors for infections were hemodialysis and prior antibiotic therapy in the previous three months for *S. maltophilia* BSI, and exposure to fluoroquinolones in the prior three months for *A. xylosoxidans* BSI. This should be considered in the management of nosocomial ICU patients, with prolonged hospitalizations, on mechanical ventilation, with extensive recent exposures to antimicrobials.
